# The Western Tibetan Vortex as an Emergent Feature of Near‐Surface Temperature Variations

**DOI:** 10.1029/2019GL085757

**Published:** 2019-12-06

**Authors:** Remco J. de Kok, Walter W. Immerzeel

**Affiliations:** ^1^ Department of Physical Geography Utrecht University Utrecht The Netherlands; ^2^ ICIMOD Kathmandu Nepal

## Abstract

Glaciers around the world are shrinking, yet in a region in northwestern High Mountain Asia (HMA), glaciers show growth. A proposed explanation for this anomalous behavior is related to the variability of the “Western Tibetan Vortex” (WTV), which correlates well with near‐surface temperatures in northwestern HMA. Using analytical formulations and ERA5 reanalysis data, we show that the WTV is the change of wind field resulting from changes in near‐surface temperature gradients in geostrophic flow and that it is not unique to northwestern HMA. Instead, we argue that net radiation is likely the main driver of near‐surface temperatures in Western HMA in summer and autumn. The decreasing strength of the WTV during summer in the twentieth century is thus likely the result of decreasing net radiation. We do argue that the WTV is a useful concept that could yield insights in other regions as well.

## Key Points

- The Western Tibetan Vortex is the result of temperature changes in northwestern High Mountain Asia
- In summer and autumn, the main driver of near‐surface temperatures in northwestern High Mountain Asia is net radiation
- The concept behind the Western Tibetan Vortex can also be applied to other regions

## 1 Introduction

Despite global warming, parts of High Mountain Asia (HMA), mainly the Western Kunlun Shan and Karakoram (WKSK), show growing glaciers, in contrast to most glaciers worldwide (e.g. Brun et al., 2017; Gardelle et al., 2012; Kääb et al., 2015). This phenomenon is known as the “Karakoram Anomaly” (Hewitt, 2005), and several possible explanations have been put forward. Glaciers in WKSK are thought to be special in their response to changes in climatic forcing, with generally heavy debris cover (Scherler et al., 2011) and low sensitivities to temperature changes (Sakai & Fujita, 2017). Furthermore, possible positive trends in winter precipitation (Cannon et al., 2015; Kapnick et al., 2014; Norris et al., 2018), as well as an increase in irrigation‐induced summer precipitation, with corresponding increases in summer cloudiness (de Kok et al., 2018) in WKSK might favor glacier growth.

Another suggestion was put forward by Forsythe et al. (2017), who found that temperatures in northwestern HMA highly correlate with the circulation around northwestern HMA at pressures between 500 and 200 hPa. This structure of correlated atmospheric variables was dubbed the “Karakoram Vortex,” or “Western Tibetan Vortex” (WTV) by Forsythe et al. (2017) and following work by Li et al. (2018, 2019), together referred to as FL17‐19 here. They propose that adiabatic heating and cooling, caused by the vortex, is the driver of temperature changes in northwestern HMA. Using this concept, they demonstrate a cooling trend in northwestern HMA, most notably in the period 1960–1980, that could help explain the Karakoram Anomaly. They attribute this cooling trend to shifts in the interplay between the westerly jet and the Indian monsoon.

Here, we offer an interpretation of the WTV that is different from FL17‐19, which is that such a vortex can be understood as a natural result of temperature changes near the surface when different years are compared. Hence, the causal relationship between temperatures and winds would be reversed compared with what was originally proposed.

## 2 Data and Methods

We used ERA5 reanalysis output (Copernicus Climate Change Service, 2017) for our analysis. Data were downloaded as monthly means, as aggregated by the KNMI climate explorer (https://climexp.knmi.nl/start.cgi). We then aggregated months, weighted by number of days per month, to obtain mean seasonal fields of temperature, wind, and surface energy fluxes for each year between 1979 and 2018. Gradients of the temperature fields are determined by the NumPy (Oliphant, 2006) gradient function. Net radiation is determined as the sum of net shortwave radiation and net longwave radiation, as aggregated by the KNMI climate explorer.

We calculated correlation coefficients for pairs of parameters based on the time series between 1979 and 2018, for each season separately. Since relationships might not be linear and residuals might not be normally distributed, we used Spearman rank correlations, computed using the SciPy package (Jones et al., 2001). For the time series, a correlation coefficient of 0.4 corresponds to a *p* = .01 and a coefficient of 0.5 corresponds to a *p* = .001. Some of our quoted correlation coefficients are higher than 0.8 (*p* < 6·10^−10^).

To determine WTV‐related correlations, averages over boxes are taken. The zonal shear index corresponding to the WTV is defined as the difference between the mean zonal wind speeds in two boxes, which are defined on either side of northwestern HMA. We assume the same horizontal extent as FL17‐19: the northern box (Box_N_) spans 40–50°N and 52.5–86.25°E and the southern box (Box_S_) spans 20–32.5°N and 52.5–93.75°E. We calculate the zonal shear index at 300 hPa, which is near the (vertical) center of the WTV. To compute mean near‐surface temperatures and calculate correlations relevant for northwestern HMA, we take a box that includes the stations discussed in Forsythe et al. (2017), which spans 35–36°N and 74–76°E (Box_K_).

We investigated the thermodynamic components of atmospheric heating to compare to Li et al. (2019). We used hourly ERA5 data of temperature and winds between 550 and 200 hPa to calculate vertical profiles of heating rates at 36°N, 75°E. We selected the period JJA for the years 1987 and 1994, which have opposite extreme values of the WTV index (Forsythe et al., 2017). Following Li et al. (2019), we separate the heating rates into adiabatic heating (*ADH*), horizontal temperature advection (*HTAD*), and diabatic heating (*DH*) as follows:

(1) $$\frac{\partial T}{\partial t}=\text{HTAD}+\mathrm{ADH}+\mathrm{DH},$$

with:

(2) $$\text{HTAD}=-V\cdot \nabla_{p}T\;$$

(3) $$\mathrm{ADH}=-\omega {\left(\frac{p}{p_{s}}\right)}^{R/C_{p}}\frac{\partial \theta }{\partial p}$$

(4) $$\mathrm{DH}=\frac{\dot{Q}}{C_{p}}$$

with *V* the horizontal wind vector, *p* pressure, *p*_*s*_ the reference pressure (1000 hPa), *R* the gas constant, *T* temperature, *ω* the vertical wind speed, *θ* the potential temperature, *Q* the heat content, and *C*_*p*_ the specific heat capacity at constant pressure. Diabatic heating is calculated from ADH and HTAD in two ways: one assuming the total heating rate is zero, as assumed by Li et al. (2019), and one where the heating rate is calculated from the temperature change ΔT over one hour, divided by the time period Δt. Both give almost identical results, which justifies the assumption made by Li et al. (2019).

An important feature of the WTV is the correlation between temperatures near the surface of northwestern HMA and wind speeds higher in the atmosphere (between 500‐200 hPa) surrounding northwestern HMA. A relation between winds and temperatures from atmospheric theory is also described by the thermal wind equation (Andrews, 2010). Under the assumption of geostrophic wind and hydrostatic balance, the vertical derivatives of zonal and meridional wind speeds *u* and *v* are described as follows:

(5) $$\frac{\partial u}{\partial \ln\left(p\right)}=-\frac{R}{f}{\left(\frac{\partial T}{\partial y}\right)}_{p}$$

(6) $$\frac{\partial v}{\partial \ln\left(p\right)}=\frac{R}{f}{\left(\frac{\partial T}{\partial x}\right)}_{p}$$

with *f* is the Coriolis parameter (*2Ω sin ϕ,* with *Ω* being the rotation rate of the Earth and ϕ the latitude), and *x* and *y* are the local zonal and meridional coordinates. The thermal wind equation thus gives a direct relation between horizontal temperature gradients, taken at constant pressure, and the vertical derivative of the wind.

## 3 Results

### 3.1 Analytical Considerations

**Figure 1:**
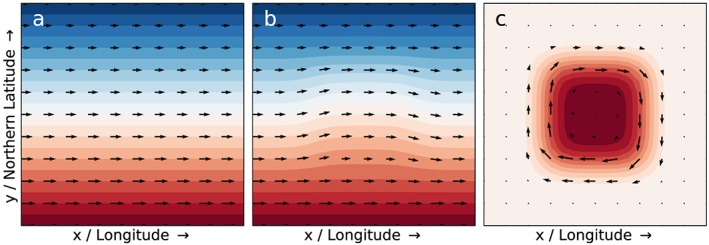
Temperature fields (shading from dark blue [cold] to dark red [warm]) and vertical gradients of horizontal wind (arrows) for (a) an initial state with a constant latitudinal temperature gradient, (b) the initial state plus a temperature perturbation, and (c) the difference between b and a.

We apply the thermal wind equation to qualitatively illustrate how a local change in temperature will change vertical wind gradients. In Figure 1a, a constant latitudinal temperature gradient is assumed in the northern hemisphere, with temperatures decreasing from equator to pole. The latitudinal dependence of the Coriolis parameter results in a latitudinal gradient of the vertical wind gradients, but there are no variations with longitude. We apply a local (positive) temperature perturbation in Figure 1b, which changes the temperature gradients and the resulting vertical wind gradients. The differences in temperature and vertical gradients of horizontal wind are illustrated in Figure 1c. It is clear that, for geostrophic winds, a change in temperatures results in a change in wind gradients that has the shape of a vortex around the region where the temperature perturbation is applied. Due to the changing Coriolis parameter, the vortex in Figure 1c is stronger near the equator than near the pole (see Eqs (5)‐(6)). Note that in neither Figure 1a nor Figure 1b a vortex is present, but it only becomes apparent when comparing the difference in wind fields.

The thermal wind equation deals with vertical gradients of horizontal wind, not with actual wind speeds. However, if the wind speeds at low altitudes are small, or if they are relatively constant over time, the actual wind speeds higher up will mostly arise from differences in the strong vertical gradient, instead of from offsets of the lower wind speeds. Hence, they will be strongly related to the vertical wind gradients shown in Figure 1. A local change in temperatures at a low altitude will then result in a vortex‐like change at higher altitudes, like the WTV.

### 3.2 Winds and Vortices in Reanalysis Data

**Figure 2:**
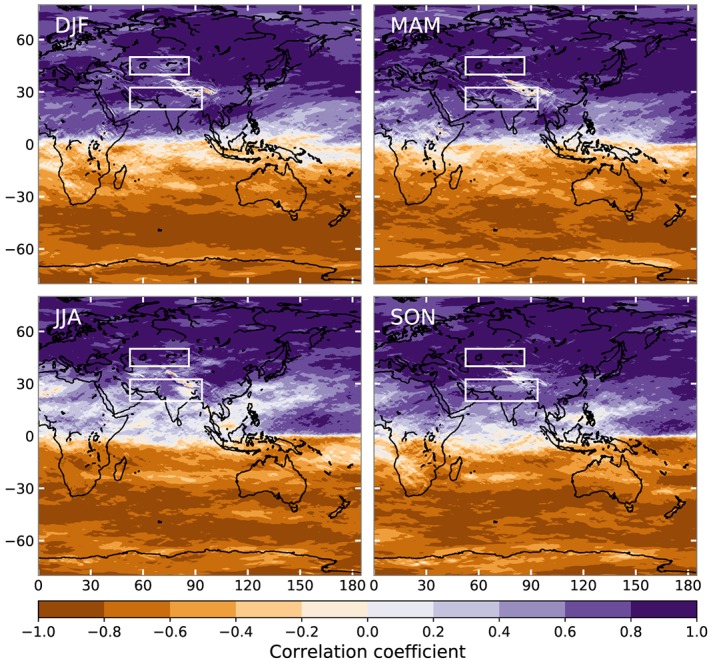
Spearman rank correlation coefficients between the latitudinal temperature gradients at 500 hPa and the zonal wind speeds at 300 hPa from ERA5 data for the four seasons. White boxes indicate Box_N_ and Box_S_.

To test whether high‐level winds and low‐level temperature gradients are indeed related in the Earth's atmosphere, we calculated correlations between latitudinal temperature gradients at 500 hPa and zonal wind speeds at the same location at 300 hPa. Figure 2 shows that this is indeed the case for most of the eastern hemisphere. The western hemisphere shows very similar behavior, but is omitted to better see HMA. The change of sign between hemispheres agrees with the change in sign of the Coriolis parameter (Eq. (5)). Notable exceptions are monsoon‐dominated regions in the tropics, and high mountains. For the high mountains, 500 hPa is close to the surface and the mountains will likely impact the winds and local temperature gradients. However, for the boxes that determine the strength of the WTV in FL17‐19, the correlation between temperature gradients and winds is generally very high. In Box_N_, the mean correlation coefficient is higher than 0.8 for MAM, JJA, and SON, whereas it is 0.68 in DJF. For Box_S_, the mean correlation coefficient is above 0.6, except for the monsoon period (JJA), where it is 0.42 (see Table S1 of the supporting information). A very similar figure is obtained for the meridional winds and longitudinal temperature gradient (see Table S1).

**Figure 3:**
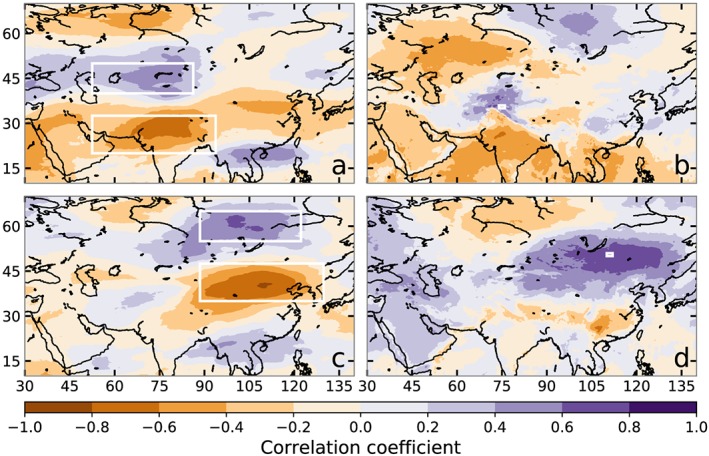
Spearman rank correlation coefficients between mean 2‐m temperatures in a small region (Box_K_ and its shifted counterpart in white) and zonal wind speeds at 300 hPa (a,c), and between the zonal shear index U_North_‐U_South_ from the large white boxes (Box_N_ and Box_S_ and their shifted counterparts) and 2‐m temperatures (b,d) during JJA. The reference boxes correspond to the ones used for WTV (a,b) and for an arbitrary other location (c,d).

Given the strong relation between temperature gradients and wind speeds in the middle and upper troposphere over much of the globe, it is likely that the vortex behavior described by the WTV is not unique to northwestern HMA. Mölg et al. (2014) already showed a similar correlation between near‐surface temperatures and wind speeds for the central Tibetan Plateau (their Figure 5b). We used ERA5 data to confirm the WTV correlations shown in FL17‐19 for ERA‐Interim and applied the same analysis to another region by shifting all boxes by a fixed amount (+15^o^ latitude and +36^o^ longitude). Figure 3 shows that there are very similar, or even stronger, correlations for this shifted vortex in JJA. We also managed to construct similarly strong “vortices” in many other locations at midlatitudes. The correlations in Box_S_ are generally stronger than in Box_N_, in line with Figure 1c. The near‐surface temperatures also correlate well with those at 500 hPa, which we used for Figure 2 (see Table S1).

### 3.3 Drivers of Near‐Surface Temperatures in Northwestern HMA

**Figure 4:**
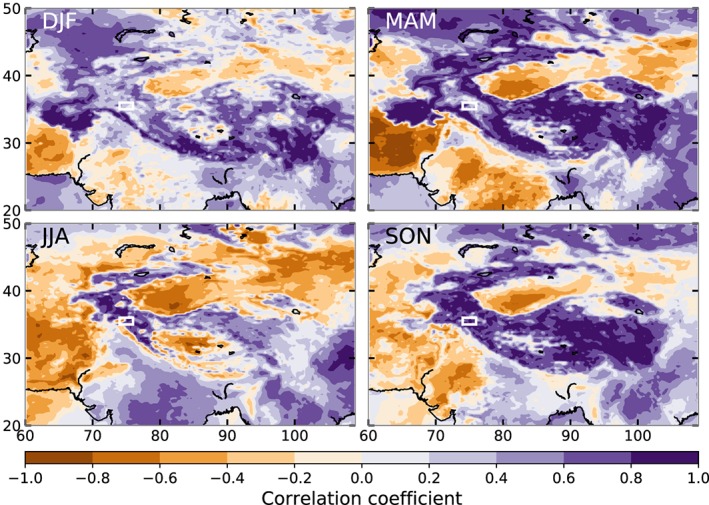
Spearman rank correlation coefficients between 2‐m temperatures and total net radiation for the four seasons. The white box shows the northwestern HMA reference box (Box_K_), where temperature correlates with the WTV in FL17‐19.

The above analysis shows that the WTV is probably not the driver of near‐surface temperatures in northwestern HMA, but that, instead, the WTV is consistent with being a response to temperature changes. The question then remains what drives the near‐surface temperatures in northwestern HMA. The temperature at the surface is driven by surface energy fluxes, such as net shortwave and longwave radiation, latent heat flux, and sensible heat flux, with the radiation terms generally being the largest (Betts et al., 1996). We performed correlations between total net radiation and near‐surface temperatures from ERA5 to see how strongly near‐surface temperatures are related to surface fluxes. Figure 4 shows a very strong correlation between total net radiation and near‐surface temperatures for much of northwestern HMA during summer and autumn, with many areas showing correlation coefficients larger than 0.8 (see Table S1). The area with high correlation in spring and autumn gives a clear outline of the entire HMA. In winter, and to a lesser extent in spring, the correlation coefficients are lower, possibly related to extensive snow cover. A snow pack complicates the relation between surface energy balance and near‐surface temperature since snow pack heating, snow melting, and evaporation also require energy. The turbulent fluxes from ERA5, and the sum of radiation and turbulent fluxes, also do not give high correlations in these periods for northwestern HMA. These low correlations are common when the largest terms of the energy balance have similar magnitudes (e.g., Litt et al., 2019). However, ERA5 data does suggest that in the ablation season of the glaciers, net radiation is the main driver of temperature fluctuations. The strong negative correlations in Figure 4 are an apparent feature of the surface energy balance in deserts, which is beyond the scope of this paper.

**Figure 5:**
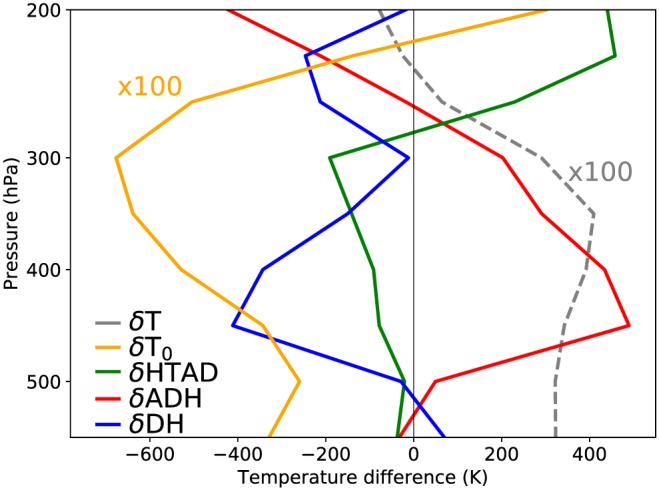
Differences in mean temperature between JJA of 1994 and 1987 above 36°N, 75°E (grey, dashed line), separated into the contributions to the temperature difference from adiabatic heating (red line), diabatic heating (blue line), horizontal temperature advection (green line), and the initial temperature difference at 1 June (orange line). Grey and orange lines are multiplied by a factor of 100 for visibility. The lowest layer (550 hPa) is close to the surface. The sum of all solid lines equals the dashed line.

These results apparently contradict those of FL17‐19, who suggest adiabatic heating is the main driver of near‐surface temperatures. Li et al. (2019) calculate differences in mean heating rates from Equation (1) for opposite WTV indices and compare these with the difference in mean temperature. They find similar patterns for adiabatic heating differences and temperature differences, and hence conclude that adiabatic heating determines WTV‐associated temperature changes. We argue that differences in the mean heating rates cannot be directly compared with mean temperature differences. For instance, a period of cooling, followed by a period of warming, will have a lower mean temperature than a period where the warming comes first, yet both periods can have identical mean heating rates. Furthermore, scaling all heating rates by a constant factor will result in a difference in mean heating rate, but not in a difference in mean temperature, since all heating terms cancel out by definition. To make a direct comparison with the mean temperature, we calculated the temperature evolution as follows:

(7) $$T\left(t\right)=T\left(t_{0}\right)+\int_{0}^{t}\frac{\partial T}{\partial \tau }\mathrm{d\tau},$$

with τ the time‐variable of integration. We calculated the contributions from each thermodynamic term by calculating the heating rates (Equations (2)–(4)) from ERA5 data and then numerically integrated them for every hour during the summers (JJA) of 2 years with opposite WTV indices (negative in 1987 and positive in 1994). We confirmed that integration errors were small. The difference in the means of these different time series (1994 minus 1987) are plotted in Figure 5. Plotted values are large, because individual terms do not often change sign during the integrated period, and hence keep adding to the integral in Equation (7). However, the different terms almost cancel out to give the total temperature difference. Figure 5 resembles Figure 10a of Li et al. (2019), except that now the different components add up to the mean temperature differences, instead of zero. Furthermore, the temperature difference in the lowest layer now comes from diabatic heating, instead of a small adiabatic heating component. This is in line with the aforementioned correlations between net radiation and near‐surface temperatures.

## 4 Discussion and Conclusions

Using atmospheric theory and ERA5 reanalysis data, we argue that the WTV is likely not the driver of near‐surface temperatures in northwestern HMA, but that the WTV is the response of the wind field to variations in near‐surface temperatures. The WTV is not a structure that can be seen in the wind field at any given time, but rather is a response that only becomes apparent when comparing different years. Such a response is also expected in other places around the globe outside the tropics and we illustrate this for a single case. Judging from the structure in Figure 1c, the presence and size of such a vortex‐like response will be related to the size of the regions where temperature changes are mutually correlated, around which the response will be visible. The size of these regions will likely be determined by the synoptic scale, by large‐scale landforms such as mountain ranges (cf. Figure 3b for northwestern HMA), and by regions of similar land cover, but this will require further study.

We show that near‐surface temperatures in northwestern HMA are highly correlated to net radiation during summer and autumn. It is therefore the likely mechanism that controls temperatures there. Furthermore, we show that the difference in temperatures at 550 hPa between 2 years of large WTV differences originates from a difference in diabatic heating instead of the adiabatic heating suggested by FL17‐19. We also tested correlations between near‐surface temperatures and vertical wind speeds at 500 hPa from ERA5 and found very low correlation coefficients (see Table S1), showing an unlikely large role for adiabatic heating near the surface. The decrease in strength of the summer WTV between 1960 and 1980, as seen by Forsythe et al. (2017), is thus the result of a decrease in net radiation and near‐surface temperatures. A cooling trend could be associated with a decrease in night‐time cloudiness in northwestern HMA, compared with daytime cloudiness (Forsythe et al., 2015).

For understanding the puzzling glacier balances in parts of Western Tibet, the WTV indicates trends in temperatures and dynamics of northwestern HMA and hence can indicate conditions that are favourable for glacier growth (Forsythe et al., 2017). The observed temperatures in the northwestern HMA do suggest a cooling trend before 1980. However, the region of correlated near‐surface temperatures associated with the WTV also includes regions that experience rapid decline of glaciers, such as Spiti Lahaul (Brun et al., 2017). Another cause of the glacier growth can thus be an increase in precipitation (e.g., Cannon et al., 2014; de Kok et al., 2018). Precipitation associated with the WTV might shed more light on this issue (Li et al., 2018), but the WTV might not be the main dynamical response to changes in precipitation, as it is for changes in temperature (Acosta & Huber, 2017; Cannon et al., 2015; Cash et al., 2015). It might be worthwhile to investigate whether direct correlations between wind fields and precipitation in a region exist in a similar way to the WTV, to gain more understanding of precipitation in northwestern HMA. To understand individual glacier behavior, detailed surface energy balance studies will give more insight into glacier melt. The surface energy balance can change across glaciers (e.g., Azam et al., 2014; Bonekamp et al., 2019; Litt et al., 2019) and might not match that of the reanalysis data, which is tuned to match observed quantities at a coarser scale. However, large‐scale trends likely influence all glaciers within a region such as WKSK to some extent. New in situ radiation measurements are needed to assess the quality of the reanalysis fluxes.

All things considered, the WTV can still be a useful concept to understand climate variability in northwestern HMA despite not being the driver of temperatures. The presence of vortex‐like structures in trends or variability of the tropospheric wind field indicate corresponding changes in temperatures. We suggest to clearly distinguish between vortices in a specific wind field and vortex‐like patterns in the variability of the wind field, such as the WTV. Direct correlations of meteorological variables with near‐surface temperatures are perhaps more insightful and intuitive than using a zonal index, but given the high correlations involved, will probably yield very similar structures. We show that an approach such as those presented in FL17‐19 can probably also be applied to other regions of interest and hence can also be of interest to researchers studying other regions.

## Supporting information



Supporting Information S1
