# Supplementary material for: The Western Tibetan Vortex as an Emergent Feature of Near‐Surface Temperature Variations
Source: Geophys Res Lett. 2019 Dec 6;46(23):14145–52. doi: 10.1029/2019GL085757 (PMC6988484; doi:10.1029/2019GL085757)
Supplement: Supplementary file 1 — Supporting Information S1 [file GRL-46-14145-s001.pdf]

AGU PUBLICATIONS

Geophysical Research Letters

Supporting Information for

The Western Tibetan Vortex as an emergent feature of near-surface temperature variations

Remco J. de Kok[1], Walter W. Immerzeel[1,2]

[1]Utrecht University, Department of Physical Geography, PO Box 80115, 3508 TC, Utrecht, The Netherlands

[2]ICIMOD, GPO Box 3226, Kathmandu, Nepal

Contents of this file

Table S1

Table S1: Mean Spearman rank correlation coefficients

Variable 1	Variable 2	Box_N	Box_S	Box_K	
U (300 hPa)	dT/dy (500 hPa)	0.69, 0.84, 0.80, 0.87	0.67, 0.59, 0.42, 0.62	-	DJF, MAM, JJA, SON (Fig. 2)
V (300 hPa)	dT/dx (500 hPa)	0.87, 0.82, 0.80, 0.83	0.67, 0.56, 0.25, 0.49	-	DJF, MAM, JJA, SON
U_N-U_s (300hPa)	T_{2m}	-	-	0.54, 0.73	JJA (Fig. 3a), JJA shifted (Fig. 3c)
U (300 hPa)	T_{2m} (Box_K)	0.36, 0.47	-0.45, -0.52	-	JJA (Fig. 3b), JJA shifted (Fig. 3d)
Net radiation	T_{2m}	-	-	0.23, 0.44, 0.46, 0.72	DJF, MAM, JJA, SON (Fig. 4)
W (500 hPa)	T_{2m}	-	-	-0.35, -0.01, 0.15, 0.08	DJF, MAM, JJA, SON
T (500 hPa)	T_{2m}	-	-	0.68, 0.61, 0.78, 0.55	DJF, MAM, JJA, SON
